# Evaluation of a radiological grading system for the early detection of total knee arthroplasties at risk for revision surgery

**DOI:** 10.1007/s00402-024-05572-3

**Published:** 2024-09-28

**Authors:** Nina Hörlesberger, Maria Anna Smolle, Lukas Leitner, Viktor Labmayr, Andreas Leithner, Patrick Sadoghi

**Affiliations:** 1 Department of Orthopaedics and Trauma, Medical University of Graz, Auenbruggerplatz 5, Graz, 8036 Austria; 2 Department of Orthopaedics and Trauma Surgery, Musculoskeletal University Center Munich (MUM), LMU University Hospital, Munich, Germany

**Keywords:** Total knee arthroplasty, Titanium-coated TKA, Survival, Revision risk TKA

## Abstract

**Introduction:**

X-rays are regularly performed after primary total knee arthroplasty (TKA). While soft tissue management and ligament tension cannot be evaluated, important information, such as inadequate component positioning and loose cement location, as well as subsequent loosening, can be detected. The aim of this study was to correlate radiological findings, referring to the radiological grading system (previously published by the same study group, henceforth abbreviated as “RGS”), with long-term outcomes and implant survival.

**Materials and methods:**

A total of 266 patients who underwent titanium-coated TKA were included. In addition to implant survival, visual analogue scale score, Tegner activity score, knee society score (KSS), Western Ontario and McMaster Universities Osteoarthritis Index, and short form-12 score as well as range of motion were evaluated. Clinical examination as well as anterior-posterior, lateral, full-length weight bearing, and patellar view radiographs were performed pre- and postoperatively, at the 3-, 6-, and 12-month postoperative follow-ups and at the final follow-up. The radiological grading system was evaluated and correlated with long-term outcome and survivorship.

**Results:**

The revision-free survival rate was 88.4% at a median follow-up of 9.8 years (IQR: 9.3–10.3 years; range: 0.1–11.8 years). Revision surgery was required in 31 TKAs (11.7%). The multivariate Cox regression model showed a significant association between an RGS score ≥ 3 deviation points (DP) and an increased risk for revision (hazard ratio: 2.092; 95% CI: 1.020–4.290; *p* = 0.044). Moreover, the KSS for pain was significantly worse in patients with a RGS score ≥ 3 DP (median, 85 [74–92] vs. 90 [80–94]; *p* = 0.007).

**Conclusions:**

This is the first study indicating that deviation in component positioning, having an inadequate long leg axis, the presence of free cement or residual bony structures on postoperative X-rays significantly correlate with TKA outcome and implant survival. Therefore RGS can be of high predicable value for the survivorship of the prosthesis.

**Level of evidence:**

Level IV – retrospective cohort study.

## Introduction

Although most implants placed during total knee arthroplasty (TKA) survive longer than ten years, the American Association of Hip and Knee Surgeons recommends biannual or annual clinical follow-up visits even for asymptomatic patients [1]. The need to reduce the frequency of such visits and the possible prediction of risk factors for revision surgery are of interest [2], as revision TKA is a serious economic burden [3, 4]. According to current calculations, the rate of revision TKAs are expected to increase from 2014 to 2030, between 78% and 182% [5].

Mathis et al. defined failure of TKA as substandard implant survival on the one hand and poor clinical outcomes on the other hand. Potential causes of failure have been defined as instability, infection, stiffness, aseptic loosening, malalignment, patellofemoral or extensor mechanism problems, and persistent pain; in most cases, early detection is cost-saving [6].

Basically, long-leg views, antero-posterior, and lateral radiographs have been mentioned to be sufficient for evaluating the coronal and sagittal alignment [7]. However, until now, no study has investigated the correlation of various parameters on direct postoperative radiographs except for malalignment with long-term clinical outcome and implant survival after primary TKA [8]. The prediction of such on direct postoperative radiographs is still not possible and therefore of high interest. Therefore, the authors previously developed a radiological grading system (henceforth abbreviated as “RGS”), taking implant malposition into consideration with regards to size, rotation, and alignment of TKA components on direct postoperative radiographs [9].

Consequently, the aim of this retrospective correlation analysis was to correlate RGS score with long-term outcomes and revision rates to detect TKA patients with a high risk of revision surgery. The hypothesis was that higher scores, indicating worse radiological results, would lead to worse clinical outcomes and higher revision rates, which would allow more personalized prospective follow-up settings.

## Materials and methods

### Study design and recruitment

All institutional rules governing clinical investigations in human subjects were strictly followed during the study; the latter was conducted in abidance with the human medical experimentation ethics document (Declaration of Helsinki of 1964 and subsequent revisions). This study was approved by the institutional review board (26–527 ex 13/14).

**Fig. 1:**
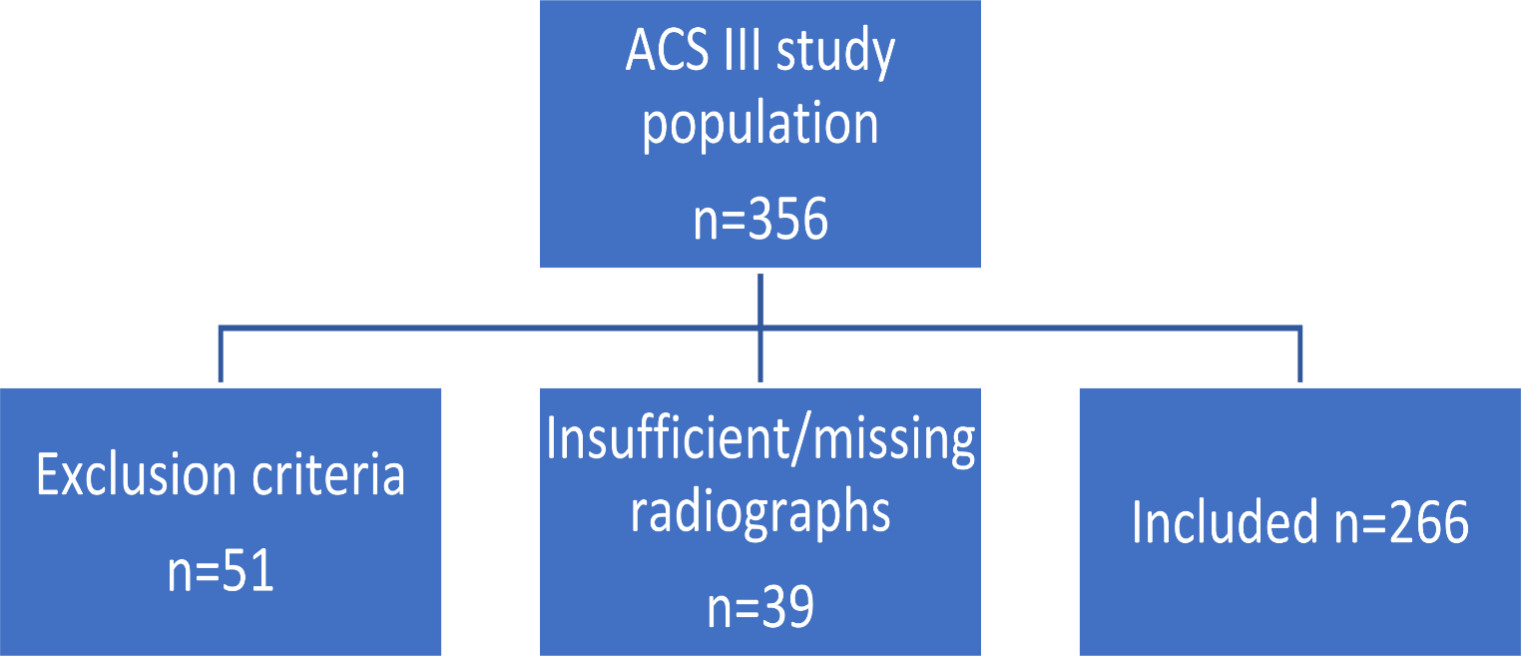
Flow Chart: a total of 356 patients were identified, after excluding 51 patients who had exclusion criteria, another 39 patients had to be excluded due to insufficient or missing radiographs. 266 patients were included in the present study

A retrospective level IV study of 266 TKAs was performed. The hospital database was searched for patients who underwent implantation of the Advanced Coated System (ACS) III (Implantcast, Buxtehude, Germany) during TKA and were followed up for a minimum of nine years (median 9.8; IQR 9.3–10.3 years). The inclusion criterion was primary TKA. Patients who had undergone revision TKA, had history of fracture or osteosynthesis of the distal femur or the proximal tibia, had undergone preoperative knee surgery except for meniscus procedures, had rheumatoid arthritis or secondary osteoarthritis after infection of the native knee joint were excluded. Furthermore patients with insufficient or missing radiographs were also excluded, e.g. over- or underexposed radiographs, were the centre of the hip can’t be defined exactly (Fig. 1, flow chart).

The ACS III (Implantcast, Buxtehude, Germany) is a mobile-bearing bi- or tri-compartmental knee prosthesis made of a CoCrMo alloy coated with TiN. The deep-dish polyethylene inlay consists of ultrahigh-molecular-weight polyethylene (UHM-WPE). Basically this implant can be used cementless and cemented. Palacos^®^ cement (Heraeus Medical, Hanau, Germany) was used. A medial parapatellar approach with single-shot antibiotic therapy with cephalosporin or lincosamide was used in all cases. All implantations were performed by or under the direct supervision of the same senior surgeon. Postoperative therapy featured full weight bearing and CPM (continuous passive motion). The visual analogue scale (VAS) score and the Tegner activity score (TAS) were assessed preoperatively and at the final follow-up. Postoperatively, we assessed the Knee Society Score (KSS), Western Ontario and McMasters Universities Osteoarthritis Index (WOMAC), and Short Form (SF)-12 score, as well as range of motion and revision rates. Time to revision was calculated from the date of surgery to the date of revision or last follow-up. Implant survivorship was defined as any surgical intervention apart from closed mobilization under anaesthesia.

Clinical examination as well as anterior-posterior, lateral, full-length weight bearing, and patellar view radiographs were performed pre- and postoperatively and at three, six, and twelve months postoperatively as well as at the last follow-up.

### The radiological grading system (RGS)

The RGS was defined to detect malposition according to the size, rotation, and alignment of the TKA components. Mechanical alignment was the desired technique in all cases. The tibial plateau was desired within varus or valgus ± 3° in the frontal plane according to mechanical alignment and the tibial slope in 5°±4° in the sagittal plane. The tibial component must not over- or underhang ± 2 mm medially and laterally, as well as anteriorly and posteriorly, according to perfect size. Inadequate sagittal alignment > 10° was defined as deviation points (DP). Furthermore, the remaining osteophytes were scored as a DP, as well as free bone cement or a tilt or shift of the patella [9]. The cut-off value for the mechanical axis was defined as 0°±3° (Table 1), as the recommended values for these parameters have been published in previous investigations [10–13].

**Table 1:** Radiological grading system (RGS)

**Tibial Component**			Score
Varus/Valgus Slope 5°		± 3°	0 or 1
		± 4°	0 or 1
Size	med-lat	± 2 mm	0 or 1
	ant-post	± 2 mm	0 or 1
**Femural Component**			
Size Hyperflexion/Hypoflexion Undercut Dorsal Osteophytes			0 or 1
		> 10°	0 or 1
		no or yes	0 or 1
		no or yes	0 or 1
**Others**			
Loose Cement Patella Deviation of Long Leg Axis		no or yes	0 or 1
		shift/tilt	0 or 1
		no or yes	0 or 1
**Overall Score**		**MIN**	**0**
		**MAX**	**11**

RGS to evaluate accuracy of primary total knee arthroplasty on direct postoperative antero-posterior and lateral X-ray. Deviations of the tibial component, femoral component, or others are classified

The measurements were performed on plain radiographs (anterior-posterior, lateral, full-length weight bearing, and patellar views). Each deviation per category counts one point on the grading system with a minimum of zero DP representing ideal implantation and a maximum of 11 DP representing the worst radiological results.

Two independent high-volume knee arthroplasty surgeons took repeated measurements independently twice with a break of one month between, and inter- and intrarater correlations were analysed.

### Statistical analysis

Statistical analyses were performed with Stata Version 16.1 for Mac (StataCorp, College Station, Texas, US). Means and medians were provided with corresponding standard deviations and interquartile ranges (IQRs). For statistical purposes, the RGS was split into two groups (Group 1: 1–2 DP; Group 2: 3–6 DP). Differences between RGS groups and functional outcome scores were assessed with Wilcoxon rank sum tests. The influence of demographic and surgery-specific parameters, as well as RGS, on the revision rate was calculated with univariate Cox regression analysis. The independent impact of variables on the revision rate was assessed with multivariate Cox regression analysis. Inter- and intraclass correlations were calculated for repeated radiological measurements. In addition, observed power was calculated according to Hoenig and Heisey [14]. A p value of < 0.05 was considered statistically significant.

## Results

Of the entire cohort (*n* = 266), 93 men (34.9%) and 173 women (65.1%) were included. The mean age at the time of surgery was 63.7 ± 8.2 years.

**Fig. 2:**
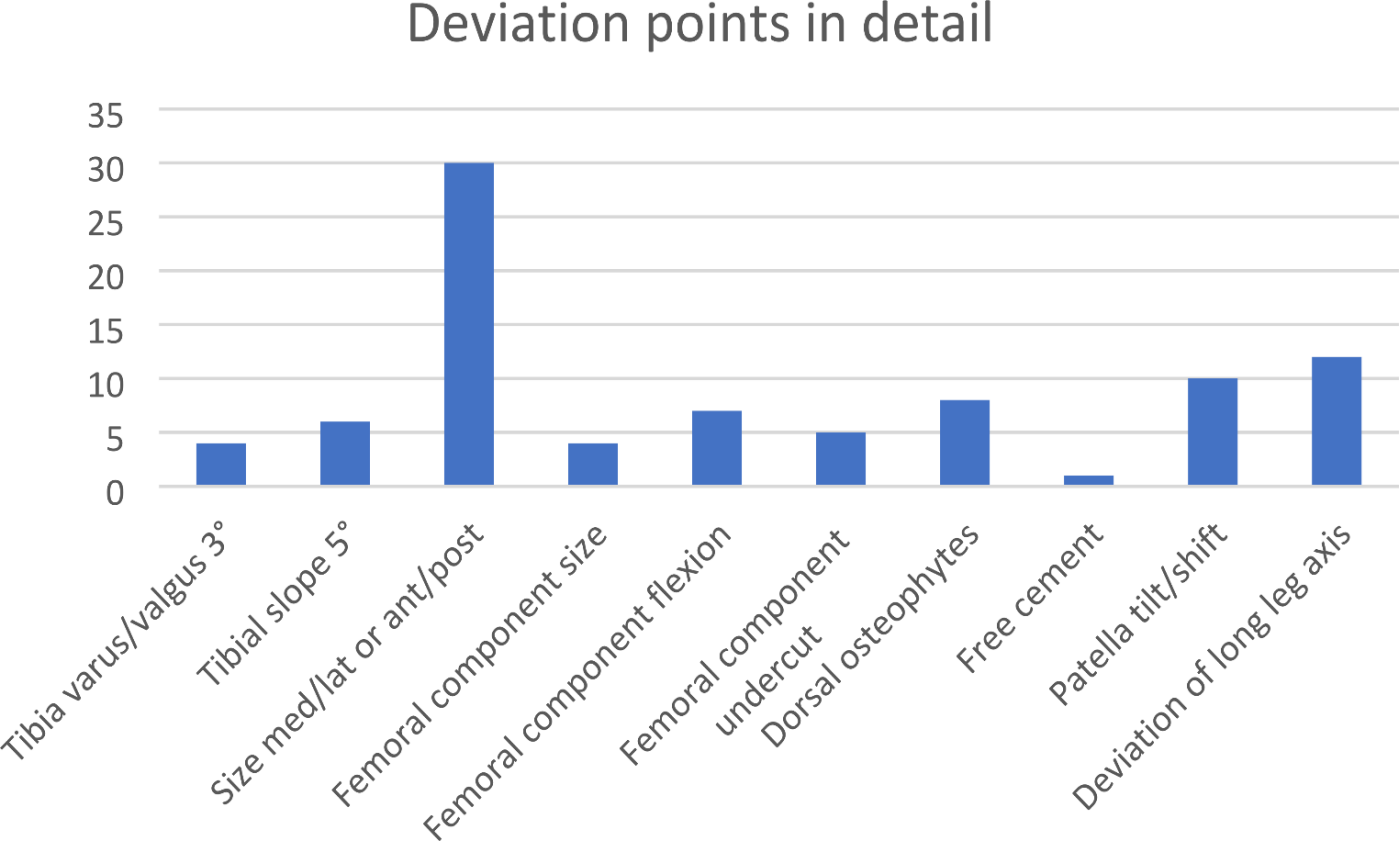
Overview of deviation points measured in the radiological grading system (RGS) to evaluate accuracy of primary total knee arthroplasty (TKA) on direct postoperative antero-posterior and lateral X-ray. Deviations of the tibial component, femoral component, or others are classified within *n* = 31 cases, which required revision surgery

The median RGS score was 2 (IQR 1–3) deviation points (DP), with the highest overall score of 6 DP (Fig. 2).

**Fig. 3:**
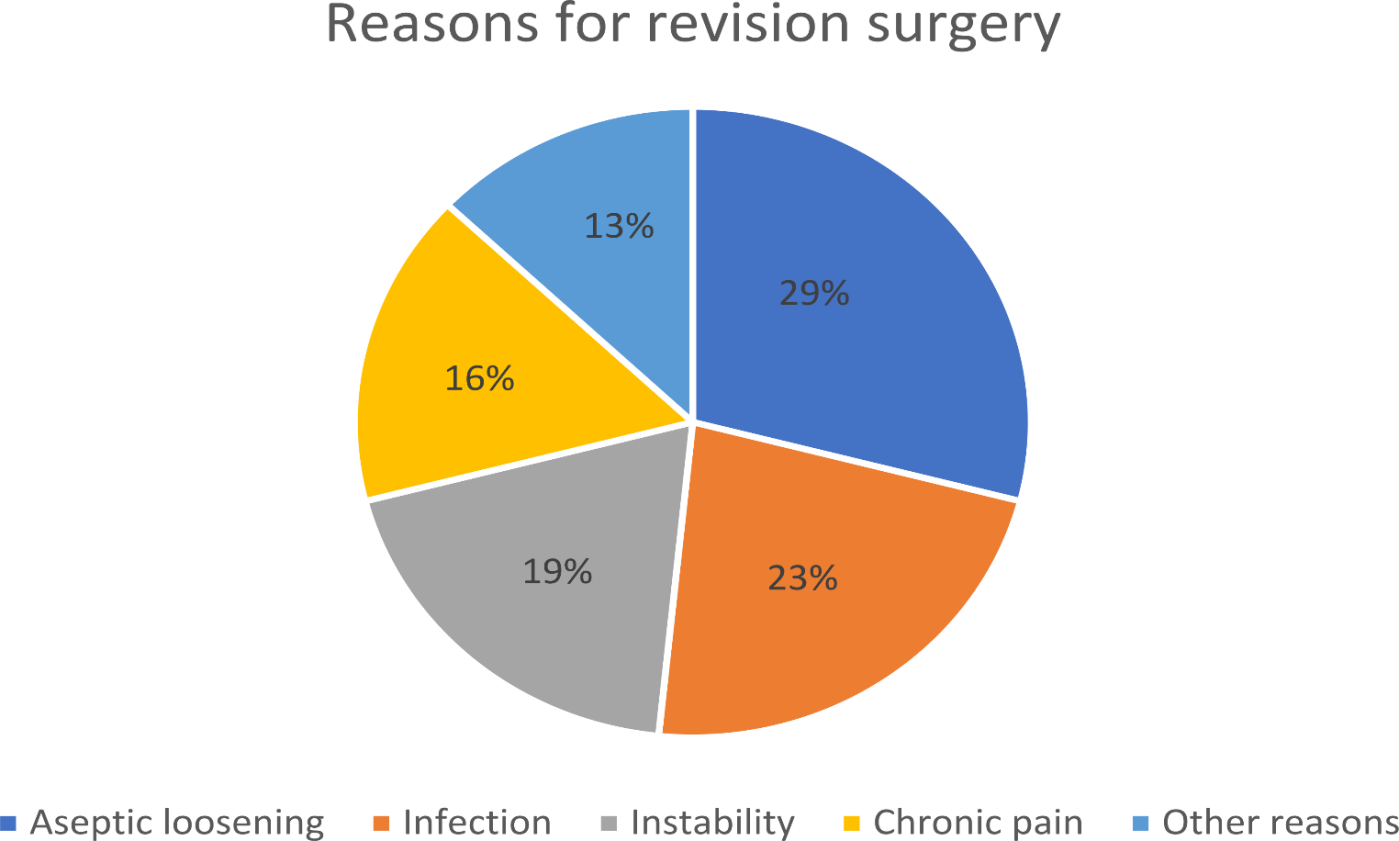
Reasons for revision surgery after implantation of *n* = 266 advanced coated system (ACS) total knee prosthesis (TKA) with a minimum follow-up of 9 years. A total of *n* = 39 cases required revision surgery: Aseptic loosening (*n* = 9), infection (*n* = 7), instability (*n* = 6), chronic pain (*n* = 5), other reasons (*n* = 4)

At a median follow-up of 9.8 years (IQR 9.3–10.3 years; range: 0.1–11.8 years), the revision rate for any cause was 11.7% (*n* = 31). Aseptic loosening was the most frequent reason for revision, occurring in nine patients (3.4%). Seven (2.6%), four (1.5%) and four (1.5%) patients underwent revision of the femoral component, tibial plateau and patella, respectively. Infection was observed in seven of 31 patients undergoing revision surgery (2.6%). In six cases (2.3%), ligament instability caused revision surgery. Chronic pain without other detectable causes was diagnosed in five of 31 cases (1.9%), and chronic reactive effusion was diagnosed in one case (0.4%). Furthermore, three patients developed periprosthetic fractures (1.1%; Fig. 3).

Closed mobilization under anaesthesia became necessary in nine patients (3.4%). Five of these patients needed revision surgery later on due to aseptic loosening or chronic pain.

### Clinical outcome parameters

KSS for pain was significantly worse in patients with a RGS score ≥ 3 DP (median, 85 [74–92] vs. 90 [80–94]; *p* = 0.007). No significant difference between RGS score groups and KSS for function (≥ RGS 3 vs. <3; median, 70 [55–90] vs. 80 [60–90], *p* = 0.413), WOMAC RGS score ≥ 3 vs. <3; median, 81.8 [72-91.7] vs. 85.6 [74.2–93.2], *p* = 0.200), postoperative TEGNER score (≥ RGS 3 vs. <3; median, 3 [2–4] vs. 3 [2–4], *p* = 0.698), SF12 physical (≥ RGS 3 vs. <3; median, 37.6 [32-43.9] vs. 37.7 [31.7–45.3], *p* = 0.312), or SF12 mental (≥ RGS 3 vs. <3; median, 50.9 [43.6–60.2] vs. 51.8 [42.9–58.6], *p* = 0.986) score was observed. Moreover, no significant difference regarding extension deficit (≥ RGS 3 vs. <3; median, 0.0 [0.0–0.0] vs. 0.0 [0.0–0.0], *p* = 0.186) or flexion deficit (≥ RGS 3 vs. <3; median, 100.0 [95.0–115.0] vs. 100.0 [95.0–115.0], *p* = 0.510) was present.

### Implant survivorship

Univariate Cox regression analysis revealed that a RGS score ≥ 3 DPs were significantly associated with higher revision rates (HR 2.258; 95% CI: 1.130–4.582; *p* = 0.024), and multivariate Cox regression analysis revealed an independent negative effect of RGS score ≥ 3 DPs on the revision rate (HR: 2.092; 95% CI 1.020–4.290; *p* = 0.044) irrespective of age at the time of surgery (*p* = 0.417) or patient sex (*p* = 0.320). No significant impact of patient age (HR 0.971; 95% CI; 0.932–1.012; *p* = 0.162) or sex (HR for males: 1.610; 95% CI 0.793–3.267; *p* = 0.187) on the revision rate was found. *With a sample size ofn = 31 for revision for any cause*, *univariate Cox regression analysis revealed* that an RGS ≥ 3 DP was significantly associated with higher revision rates (HR 2.258; 95% CI: 1.130–4.582; *p* = 0.024), *which was large enough that post hoc power analysis revealed over 80% at a p value less than 0.05.* Inter- and intraclass correlations revealed substantial agreement rates with values greater than 0.8 each.

## Discussion

This study shows that radiological deviation according to the presented radiological grading system of direct postoperative X-rays significantly correlates with the TKA outcome and implant survival at a minimum follow-up of nine years and that the presented radiologic grading system is a useful tool to detect patients at risk for revision surgery before clinical manifestation. In addition, this is the first long-term outcome study of TKA with the ACS III prosthesis.

The three major causes for failure of TKA are aseptic loosening, instability, and infection [6, 15], which is in line with the presented dataset of this cohort study. According to most surgeons, malalignment diminishes the likelihood of achieving a good clinical outcome and the longevity of TKA implants [15]. Malposition of the femoral component has been reported to be associated with tibial aseptic loosening. The authors concluded that the origin of aseptic loosening of the tibial component was the misrotated femoral component, resulting in a deviated long leg axis [16]. This is in line with the recent results of this study, as nine out of nine patients with aseptic loosening showed deviation points either in a malrotation of the femoral component or a deviated long leg axis.

Survival of radiologically malrotated total knee arthroplasty implants with respect to prosthetic and long leg alignment has been investigated in numerous articles in the past [17–19]. Ritter et al. demonstrated that people with preoperative varus greater than 8 degrees and valgus greater than 11 degrees have a 2.3% greater risk of failure and that substantial correction of only one component to produce neutral alignment increased the risk of failure in 5342 and 6070 cases [17, 18]. In addition, it was found that failure was most evident in cases of a tibial alignment < 90 degrees and a femoral component greater than eight degrees of valgus [18].

Kim et al. [19] added sagittal and axial alignment and stated that surgeons should aim for overall anatomical knee alignment at an angle of 3-7.5° valgus, femoral component alignment at 2–8.0° valgus, femoral sagittal alignment at 0–3°, tibial coronal alignment at 90°, tibial sagittal alignment at 0–7°, femoral rotational alignment at 2–5° external rotation, and tibial rotational alignment at 2–5° external rotation. This is in line with an investigation by our own study group, which evaluated the impact of the tibial slope on the outcome and range of motion [10]. In 2018, Lee et al. [16] demonstrated that loosening was most evident on the tibial plateau in 1299 cases of excessive varus alignment. However, the concept of constitutional varus has not been included in these investigations nor revealed sufficient long-term survival outside of these boundaries in clinical studies or survival analyses [20].

The impact of free bone cement has been evaluated in unicompartmental knee arthroplasty (UKA) but has not been studied in TKA with respect to long-term outcome and survival [21]. Therefore, the presented radiological grading system, including sizing of the prothesis, overall alignment and specific tibial and femoral alignment, remaining osteophytes, alignment of the patella, and free bone cement, has been evaluated in parts but not summarized under standardized settings, as presented here.

With respect to clinical outcome, Awengen et al. demonstrated that after TKA, symptomatic patients showed significantly more flexed and more internally rotated components than asymptomatic patients [22], which is further underlined by Mathis et al., who published typical patterns in SPECT/CT in patients after TKA. Authors found more flexed femoral components, a more varus femoral component, and more posterior slope as significant causes of pain after TKA [23]. In the present study, all five patients with persisting pain after revision surgery showed a patella tilt/shift, a tibial slope deviation, and/or inadequate size of one component.

Causes of instability, flexion/extension gap mismatch, component malposition, ligament insufficiency, extensor mechanism insufficiency, component loosening and global instability have been mentioned [24]. Six patients received revision surgery due to instability in this study, mainly caused by an incorrect component size or leg axis deviation, while component loosening was evaluated separately.

Grimberg et al. investigated the risk of early prosthetic joint infection in ceramic-coated TKA prostheses. Analogous to the present data, Grimberg et al. found no significantly reduced risk of periprosthetic joint infection in ceramic-coated implants [25].

Sambandam et al. recently reported that patients who undergo manipulation under anaesthesia are at higher risk of revision [26], which can be confirmed by the present study, where five out of nine patients had revision surgery later on. The main finding of the present study is that there exists a significant correlation between a worse KSS for pain as well as a higher revision rate with higher RGS score (≥ 3 DP) (*p* = 0.007 for KSS pain, *p* = 0.0224 for revision surgery).

The following limitations of this study must be underlined. First, the level of evidence is low due to its retrospective nature. However, this common study design and long-term follow-up do not necessarily change the outcome of the correlation analysis. In addition, functional outcome not only relies on preoperative conditions, surgical accuracy and postoperative care, including physiotherapy and rehabilitation but also on overall health and circumstances, such as COVID-19 pandemic, which might have changed over the course of the long follow-up. We want to underline that the revision rate after ten years is higher than reported in the literature [Teeny SM, York SC, Mesko JW, Rea RE. Long-term follow-up care recommendations after total hip and knee arthroplasty: results of the American Association of Hip and Knee Surgeons’ member survey [1]. This difference of 1 to 2 patients out of 100 over one decade is clinically minimal and further underlines the credibility of our reporting. Furthermore the titanium coating of the ACS TKA system is not standard and therefore a potential limitation regarding transferability to other TKA systems [27]. As another limitation, we chose the median RGS as the cut-off value.

The strength of this study is that the presented work relies on a previously published RGS, which already had been associated with specific outcome analysis [9], and that the evaluation of deviations was analysed using intra- and interobserver correlations.

Overall, this study could identify risk factors for inferior outcomes and revision according to direct postoperative X-rays on a grading system. This is clinically relevant as surgeons may use the RGS in order to meticulously evaluate all parameters of direct postoperative X-rays and then incorporate their own findings in future cases. Furthermore, based on the RGS, follow-up examinations could be planned more individually, e.g., in cases of low revision risk and broader intervals, which would reduce costs. Using this system, the outcome of total knee replacement will be improved in the future.

## Conclusions

The present study showed that radiological deviation according to our presented grading system of direct postoperative X-rays significantly correlates with the clinical outcome of TKA and survival of related prostheses during 9 years of follow-up. We believe that it represents a useful tool to detect patients at risk for revision surgery before clinical manifestation.

## Data Availability

Data is available upon reasonable request from the corresponding author.

## References

[1] Teeny SM, York SC, Mesko JW, Rea RE (2003) Long-term follow-up care recommendations after total hip and knee arthroplasty: results of the American Association of hip and knee surgeons’ member survey. J Arthroplasty 18(8):954–962 14658097 10.1016/j.arth.2003.09.001

[2] Hightower CD, Hightower LS, Tatman PJ, Morgan PM, Gioe T, Singh JA (2016) How often is the office visit needed? Predicting total knee arthroplasty revision risk using pain/function scores. BMC Health Serv Res 16(1):429 27553056 10.1186/s12913-016-1669-y PMC4995795

[3] Livshetz I, Sussman BH, Papas V, Mohamed NS, Salem HS, Delanois RE et al (2023) Analyzing the Burden of Revision Total Knee Arthroplasty in the United States between 2009 and 2016. J Knee Surg 36(2):121–131 34237780 10.1055/s-0041-1731324

[4] Delanois RE, Mistry JB, Gwam CU, Mohamed NS, Choksi US, Mont MA (2017) Current Epidemiology of Revision Total Knee Arthroplasty in the United States. J Arthroplasty 32(9):2663–2668 28456561 10.1016/j.arth.2017.03.066

[5] Schwartz AM, Farley KX, Guild GN, Bradbury TL (2020) Jr. Projections and epidemiology of revision hip and knee arthroplasty in the United States to 2030. J Arthroplasty 35(6S):S79–S85 32151524 10.1016/j.arth.2020.02.030 PMC7239745

[6] Mathis DT, Hirschmann MT (2021) Why do knees after total knee arthroplasty fail in different parts of the world? J Orthop 23:52–59 33456216 10.1016/j.jor.2020.12.007 PMC7797486

[7] Rohner E, Heinecke M, Matziolis G (2021) Diagnostic algorithm in aseptic TKA failure - what is evidence-based? J Orthop 24:248–253 33854292 10.1016/j.jor.2021.03.006 PMC8039505

[8] Kumar N, Yadav C, Raj R, Anand S (2014) How to interpret postoperative X-rays after total knee arthroplasty. Orthop Surg 6(3):179–186 25179351 10.1111/os.12123 PMC6583264

[9] Hoerlesberger N, Glehr M, Amerstorfer F, Hauer G, Leithner A, Sadoghi P (2021) Residents’ learning curve of total knee arthroplasty based on Radiological Outcome parameters: a retrospective comparative study. J Arthroplasty 36(1):154–159 32839061 10.1016/j.arth.2020.07.045

[10] Kastner N, Sternbauer S, Friesenbichler J, Vielgut I, Wolf M, Glehr M et al (2014) Impact of the tibial slope on range of motion after low-contact-stress, mobile-bearing, total knee arthroplasty. Int Orthop 38(2):291–295 24346515 10.1007/s00264-013-2242-5 PMC3923942

[11] Longstaff LM, Sloan K, Stamp N, Scaddan M, Beaver R (2009) Good alignment after total knee arthroplasty leads to faster rehabilitation and better function. J Arthroplasty 24(4):570–578 18534396 10.1016/j.arth.2008.03.002

[12] Abdel MP, Ollivier M, Parratte S, Trousdale RT, Berry DJ, Pagnano MW (2018) Effect of postoperative mechanical Axis Alignment on Survival and functional outcomes of modern total knee arthroplasties with cement: a Concise follow-up at 20 years. J Bone Joint Surg Am 100(6):472–478 29557863 10.2106/JBJS.16.01587

[13] Rand JA, Coventry MB (1988) Ten-year evaluation of geometric total knee arthroplasty. Clin Orthop Relat Res. (232):168–173 3383484

[14] Hoenig J, Heisey D (2001) The abuse of power: the Pervasive Fallacy of Power Calculations for Data Analysis. Am Stat 55:19–24

[15] Sadoghi P, Liebensteiner M, Agreiter M, Leithner A, Bohler N, Labek G (2013) Revision surgery after total joint arthroplasty: a complication-based analysis using worldwide arthroplasty registers. J Arthroplasty 28(8):1329–1332 23602418 10.1016/j.arth.2013.01.012

[16] Lee BS, Cho HI, Bin SI, Kim JM, Jo BK (2018) Femoral component Varus Malposition is Associated with tibial aseptic loosening after TKA. Clin Orthop Relat Res 476(2):400–407 29389790 10.1007/s11999.0000000000000012 PMC6259714

[17] Ritter MA, Davis KE, Davis P, Farris A, Malinzak RA, Berend ME et al (2013) Preoperative malalignment increases risk of failure after total knee arthroplasty. J Bone Joint Surg Am 95(2):126–131 23324959 10.2106/JBJS.K.00607

[18] Ritter MA, Davis KE, Meding JB, Pierson JL, Berend ME, Malinzak RA (2011) The effect of alignment and BMI on failure of total knee replacement. J Bone Joint Surg Am 93(17):1588–1596 21915573 10.2106/JBJS.J.00772

[19] Kim YH, Park JW, Kim JS, Park SD (2014) The relationship between the survival of total knee arthroplasty and postoperative coronal, sagittal and rotational alignment of knee prosthesis. Int Orthop 38(2):379–385 24173677 10.1007/s00264-013-2097-9 PMC3923934

[20] Howell SM, Shelton TJ, Hull ML (2018) Implant survival and function ten years after kinematically aligned total knee arthroplasty. J Arthroplasty 33(12):3678–3684 30122435 10.1016/j.arth.2018.07.020

[21] Hauptmann SM, Weber P, Glaser C, Birkenmaier C, Jansson V, Muller PE (2008) Free bone cement fragments after minimally invasive unicompartmental knee arthroplasty: an underappreciated problem. Knee Surg Sports Traumatol Arthrosc 16(8):770–775 18516590 10.1007/s00167-008-0563-5

[22] Awengen R, Rasch H, Amsler F, Hirschmann MT (2016) Symptomatic versus asymptomatic knees after bilateral total knee arthroplasty: what is the difference in SPECT/CT? Eur J Nucl Med Mol Imaging 43(4):762–772 26666238 10.1007/s00259-015-3278-0

[23] Mathis DT, Tschudi S, Amsler F, Hauser A, Rasch H, Hirschmann MT (2022) Correlations of typical pain patterns with SPECT/CT findings in unhappy patients after total knee arthroplasty. Knee Surg Sports Traumatol Arthrosc 30(9):3007–3023 33864469 10.1007/s00167-021-06567-y PMC9418274

[24] Song SJ, Detch RC, Maloney WJ, Goodman SB, Huddleston JI (2014) 3rd. Causes of instability after total knee arthroplasty. J Arthroplasty 29(2):360–364 23896358 10.1016/j.arth.2013.06.023

[25] Grimberg AW, Grupp TM, Elliott J, Melsheimer O, Jansson V, Steinbruck A (2021) Ceramic coating in cemented primary total knee arthroplasty is not Associated with decreased risk of revision due to early prosthetic joint infection. J Arthroplasty 36(3):991–997 33012599 10.1016/j.arth.2020.09.011

[26] Sambandam S, Mounasamy V, Wukich D (2022) Patients undergoing manipulation after total knee arthroplasty are at higher risk of revision within 2 years. Eur J Orthop Surg Traumatol 32(1):145–150 33760999 10.1007/s00590-021-02943-z

[27] Utzschneider S, Harrasser N, Sadoghi P, Weber P, Schroder C, Pietschmann MF et al (2010) Crosslinked polyethylene in knee arthroplasty: a simulator study evaluating the positive influence on the tribocontact area in the fixed-bearing knee. Arch Orthop Trauma Surg 130(11):1419–1424 20690024 10.1007/s00402-010-1159-3

